# Sensor Network Configuration Learning for Maximizing Application Performance

**DOI:** 10.3390/s18061771

**Published:** 2018-06-01

**Authors:** Joel Helkey, Lawrence Holder

**Affiliations:** School of Electrical Engineering and Computer Science, Washington State University, Pullman, WA 99164, USA; jhelkey@wsu.edu

**Keywords:** wireless sensor network, network simulation, maximizing performance, iterative improvement

## Abstract

Numerous applications rely on data obtained from a wireless sensor network where application performance is of utmost importance. However, energy usage is also important, and oftentimes, a subset of sensors can be selected to maximize application performance. We cast the problem of sensor selection as a local search optimization problem and solve it using a variant of stochastic hill climbing extended with novel heuristics. This paper introduces sensor network configuration learning, a feedback-based heuristic algorithm that dynamically reconfigures the sensor network to maximize the performance of the target application. The proposed algorithm is described in detail, along with experiments conducted and a scalability study. A quick method for launching the algorithm from a better starting point than random is also detailed. The performance of the algorithm is compared to that of two other well-known algorithms and randomness. Our simulation results obtained from running sensor network configuration learning on a number of scenarios show the effectiveness and scalability of our approach.

## 1. Introduction

Sensor applications are deployed by using data obtained from a wireless sensor network (WSN), and this approach is applicable in many domains. For example, it can be used in smart homes to detect individual movement and classify the task in which the individual is engaged [1]; in this case, the goal of the application is to maximize recognition accuracy. However, giving an AI application too much data from too many attributes can cause its performance to decrease.

Sensors in a WSN typically run on batteries. Constantly gathering all the data from all the sensors can be cost prohibitive and often unnecessary. Hence, many times, a subset of sensors must be selected to maximize application performance, allowing the possibility of turning off unnecessary sensors to save the energy needed to power them. Finding the optimal subset of sensors, the so-called sensor selection problem, is known to be NP-hard [2]. A naive approach would be to exhaustively try all possible combinations of sensors; however, such an approach is infeasible as the state space increases exponentially with the number of sensor nodes, and the time to evaluate each combination in a real-world environment would be prohibitive.

There are two main ways to solve the sensor selection problem: exact methods (e.g., convex/non-convex optimization [3,4]) and heuristic methods (covered in Section 2). Exact methods rely on being able to know or approximate the reward Equation, and we reject the notion that over a large exponential state space, this can be done a priori with complete disregard for the performance of the application running on the network. Hence, our approach to this problem focuses on finding a heuristic solution. We cast the problem of sensor selection as a local search optimization problem and solve it with the application of a novel variant of stochastic hill climbing [5] that has been extended with novel heuristics especially relevant to sensor network configurations.

### 1.1. Problem Scenario

The scenario we address with this work is as follows: given a set *N* of sensor network nodes, a configuration *c* specifying the state of each node as “on” or “off” and a reward Equation R(c) that measures the performance of an application using the data collected from the network as configured according to *c*, find the configuration $c^{*}$ maximizing *R*.

We make four assumptions about the scenario. First, network nodes are stationary. Second, the data sending rate is fixed, and nodes are either on or off. Third, the application must be capable of reporting its performance in a meaningful way, such as accuracy on a scale of 0%–100%, for an AI application. Finally, the reward Equation does not change while sensor network configuration learning (SNCL) is running. Future work will need to detect transitions that signal a change in the reward Equation and adapt the algorithm to react more quickly to that situation.

Since most sensor networks are ad hoc, meaning nodes can serve as routers, as well as just sources, then the second assumption may have far-reaching effects. Turning off a node (that also serves as a router) could cause other nodes’ sensor data to fail to reach the sink. In our experiments with power consumption estimates in network simulators [6], we implemented a scheme by which nodes that were turned “off” were actually put in a sleep state, except that they would wake for a few seconds each minute to check for and route a new configuration and then go back to sleep or turn on. In this way, our method can still turn on nodes even when their sensor data may not have a route to the server. If this degrades application performance, then eventually, our method will configure the network appropriately so that sensor data critical for maximum performance can be collected. Therefore, we feel confident that our algorithm will be able to deal with this aspect of performance, as well. If turning on a node increases application performance, it does not matter if that specific node’s data contributes to the increase or if it is routed sensor data that cause the increase.

### 1.2. Contributions

This paper introduces sensor network configuration learning (SNCL), a feedback-based learning algorithm that takes feedback from the application performance along with the current network state and dynamically reconfigures the WSN with the goal of learning a configuration that will maximize the performance of the target application. In particular, we use the idea of iterative improvement with the goal of finding the optimal or the best configuration in the time allowed, but our approach is more specialized to sensor network configurations.

The simulation results using a variety of reward Equations show that SNCL performs better than pure randomness, a standard genetic algorithm [7] and probabilistic selection [8], a recently published sensor selection algorithm. In particular, SNCL is able to find a configuration that maximizes application performance in all scenarios; whereas pure randomness and genetic algorithm are never able to find the maximum within the given time frame, and probabilistic selection only finds it in two scenarios due to trying all nodes on as a first step.

The rest of this paper is structured as follows. Section 2 presents related work. Section 3 provides the details of SNCL. Section 4 enumerates the algorithms. Section 5 presents the simulation results. Section 6 discusses these results. Finally, Section 7 contains concluding remarks and future research directions.

## 2. Related Work

Heuristic methods for sensor selection have been widely researched in a variety of areas. However, when deciding which sensors should be used, actual application performance is rarely considered.

For example, Joshi and Boyd [9] propose a convex optimization-based heuristic for approximately solving the sensor selection problem where each sensor produces a signal defined by a linear Equation plus additive noise. The measurement noise sources are non-correlated identically distributed zero-mean Gaussian random variables. Their goal is to choose a set of sensors that will minimize the determinant of the estimation error covariance matrix.

Damuut et al. [10] apply T-norm fuzzy logic to the problem, with their formulation taking into consideration sensor energy reserve, distance to the sink and sensor readings. Shih et al. [11] propose a scheme based on coverage, whereby full coverage is achieved by identifying redundant sensors via Voronoi diagrams and turning them off. Buczak et al. [12] use a genetic algorithm to select sensors for maximizing the accuracy of target tracking and minimizing the power consumption of the sensor network. In the fitness Equation for estimating the position errors, they do not use the actual target position; rather, they use an approximate target position predicted by the tracker. Damuut and Gu [13] also consider a genetic algorithm approach where the objective Equation is specified in advance. Hence, application performance is not considered. Gupta et al. [14] present a stochastic sensor selection algorithm that chooses sensors randomly according to a probability distribution with the goal of minimizing expected steady state error covariance. However, they use the expected error rather than the actual error. Zhang et al. [15] address the specific problem of maximizing network lifetime by balancing energy usage and data collection frequency in a concentric rings topology, but do not consider the case where maximizing data collection frequency may result in sub-optimal application performance.

For more closely-related work in this strand of the literature, application performance is important for determining sensor selection. For example, Wendt et al. [16] use a semantics-driven approach for a wearable sensing system. For a medical shoe, the raw data come from accelerometers and pressure sensors, while the semantic information derived from those are gait characteristics, the speed of impact, and so on. The idea is to keep the best predicting sensors. Zhang and Zhang [17] propose a decentralized approach to the specific application of target classification by fusing the decisions of individual nodes and thus reducing power consumption through less communication.

The work closest to ours, Xu and Potkonjak [8], proposes iterative selection and probabilistic selection for the sensor selection problem and demonstrates how this works with a medical shoe. As with the previous paper, they find that semantic information is more important than focusing on the accuracy of the raw sensor data. The authors assume that the best prediction accuracy (application performance) occurs when all sensors are on. However, their algorithms only make one pass in creating a working configuration.

Our work differs from Xu and Potkonjak in several ways. Most importantly, we generalize the feedback to application performance and do not specifically focus on the predictability of factors as would be appropriate for machine learning types of applications. Moreover, we do not assume that the highest application performance occurs when all the sensors are on; in fact, it could be any subset. We further show that our approach is superior to Xu and Potkonjak’s in our experimental results.

## 3. The SNCL Algorithm

We developed the SNCL Algorithm 1, which dynamically reconfigures the wireless sensor network by using the feedback from application performance to learn a configuration that will maximize the performance of the target application. SNCL consists of two main components, a variant of stochastic hill climbing for navigating the large state space of possible network configurations and a quick start algorithm for rapidly determining a good starting configuration. See Table 1 for algorithm symbol definitions.

### 3.1. Search Method

Stochastic hill climbing typically evaluates all neighbors and then chooses randomly among them, weighted by their evaluation. However, since it is known that exhaustively trying all possible configurations is infeasible, our approach using SNCL does not try all neighbors, but more intelligently selects the neighbors to evaluate based on the learned trajectory of the reward Equation. To extend stochastic hill climbing, we define who the neighbors are and what constitutes an uphill move.

**Algorithm 1** Sensor network configuration learning.1:**procedure** SNCL(*N*, *T*)2: *N*: Number of nodes3: *T*: Number of iterations   4: H←N5: **if** Quick start option to be used **then**6:  $c_{current}\leftarrow$ QuickStartConfiguration(*N*)7: **else**8:  $c_{current}\leftarrow$ random configuration9: **end if**10: $c_{max}\leftarrow c_{current}$11: ⊳ Explore with directional search12: i←113: h←H14: $d_{last}\leftarrow$ last direction (randomly initialized)15: **while** i≤T **do**   16:  $h\leftarrow h-\frac{\left(H-1\right)}{\left(T-1\right)}$17:  **if** iteration i−1 increased the reward **then**18:   $c_{current}\leftarrow$ RandomConfig($c_{max},h,d_{last}$)19:  **else**20:   $c_{current}\leftarrow$ RandomConfig($c_{max},h,\neg d_{last}$)21:  **end if**22:  wait for reward feedback23:  $c_{max}\leftarrow \max\left(c_{max},c_{current}\right)$24:  update $d_{last}$25:  i←i+126: **end while**27: **return** $c_{max}$28:**end procedure**


The idea of this algorithm is to keep a single current state and iteratively try to improve it. The algorithm starts with an initial configuration and searches a neighborhood of that configuration for one that will result in higher application performance. The neighborhood of configuration *c* is defined as a set of configurations that are in some sense close to *c*, where the Hamming distance is used as a closeness measure. We use a linearly decreasing Hamming distance and directionality (based on previous application performance) to define a neighborhood. The neighborhoods are never intentionally fully explored by this algorithm as that is deemed too costly in terms of time and taking into consideration the exponentially large search space.

Directionality is determined by the action of turning configuration bits on and off. Turning bits on is one direction, and turning bits off is the opposite direction. If turning on a bit in the last iteration caused an increase in application performance, then we want to continue in the direction of turning on bits. Otherwise, we go in the direction of turning bits off.

At each iteration, the algorithm randomly picks a configuration from the neighborhood as constrained by Hamming distance and directionality. If application performance increases, it replaces the current configuration, and the algorithm continues to move in the same direction. Otherwise, the current configuration stays the same, and the direction is reversed. The algorithm continues to iterate over the exploration time window.

Specifically, Algorithm 1 first sets the maximum Hamming distance *H* to the number of nodes *N* in the network (Line 4). In Lines 5–10, configuration $c_{max}$ is initialized to either a completely random configuration or one determined by the QuickStartalgorithm (described later). Lines 12–14 initialize the search loop iteration number *i* (that runs from 1 to *T*), the desired Hamming distance *h* between $c_{max}$ and the next configuration tried and the direction $d_{last}$ the algorithm moved in the last iteration (turned nodes on, or turned nodes off). The while loop in Lines 15–26 tries *T* times to find the best ($c_{max}$) configuration, which is ultimately returned. The while loop first computes the desired Hamming distance *h* of the next configuration $c_{current}$ to try. Line 16 ensures that the value of *h* is reduced from *H* –1 uniformly over the span of iterations *T*. Lines 17–21 generate $c_{current}$ as a new random configuration that is a Hamming distance *h* from $c_{max}$ (or the largest Hamming distance possible and less than *h*; see Algorithm 2). The new configuration is achieved by either turning nodes on or turning nodes off, depending on whether the direction $d_{last}$ from the previous iteration increased performance. Line 22 represents the process of actually reconfiguring the network and waiting to evaluate application performance based on this new configuration. This process will vary depending on the application. If the new configuration $c_{current}$ is better, then $c_{max}$ is set to $c_{current}$, and the direction $d_{last}$ is updated to be the direction resulting in this better configuration (Lines 22–23).

The recommendation for setting *T* (the number of exploration iterations) is $10\times N^{2}$. However, if you know the maximum performance value for the application, then you can stop the algorithm once that is reached (and restart it, if performance ever drops).

### 3.2. Random Configuration

The SNCL algorithm needs to have a way to obtain a random configuration that is a given Hamming distance away from the current configuration. An obvious approach to solving this problem would be to use a brute-force algorithm, where all possibilities are enumerated, then one would be chosen at random. However, the brute-force approach does not scale as the number of nodes increases, because the number of possible configurations increases exponentially. Therefore, an O(n) algorithm was created to address this issue, and we called it the random configuration algorithm.

The random configuration Algorithm 2 returns a random configuration that is Hamming distance *h* away from configuration *c* in the desired direction *d*. If *h* cannot be accomplished, but a move in the desired direction is possible, then the maximum achievable Hamming distance is used. If it is not possible to move in the desired direction, then a random restart is performed by returning a completely random configuration.

Specifically, Algorithm 2 begins by initializing the maximum achievable Hamming distance $h_{max}$ to the given desired distance *h* and setting *m* to the simple majority of *h* (Lines 5–6). This majority value will be used to ensure that a majority of the bit flips are in the desired direction. Lines 7–11 determine the number of bits *B* that can be flipped in the desired direction. If B=0, then we cannot move at all in the desired direction and so return a random configuration (Lines 12–15, i.e., it reached a local maximum, so do a random restart). If B>0, but B<m, then we cannot flip a majority of the bits in the desired direction, so the maximum achievable Hamming distance is reduced appropriately to $h_{max}=2B-1$, whose majority is then m=B. Then, in Lines 20–24, a majority of the flippable bits are flipped in the desired direction. Line 25 computes the number of bits *r* remaining to be flipped in order to achieve a distance of $h_{max}$. Lines 26–27 choose a random number $F_{01}$ in [0,r) of previously-unflipped bits to flip from zero (off) to one (on), recording in *a* the actual number of bits flipped (there may not have been enough unflipped zero bits to flip). Finally, Lines 28–29 determine the number of previously unflipped one bits that need to be flipped to zero (off), in order to achieve the $h_{max}$ Hamming distance from the input  configuration.

As an example, suppose the original configuration is *c* = 11111100, with a desired Hamming distance of h=5 and a desired direction *d* = on. Clearly, the desired distance cannot be achieved in the desired direction, i.e., there are only two bits (B=2) that can be flipped from zero to one. Thus, the algorithm resets $h_{max}=2B-1=3$ and majority m=2. The majority bits are then flipped, resulting in the configuration 11111111. One additional bit ($r=h_{max}-m$) must be flipped to achieve the new $h_{max}$. Since all bits are one, then $F_{01}=0$, $F_{10}=1$, and one of the original one bits, chosen at random, is flipped to zero, resulting in one of six possible final configurations: 01111111, 10111111, 11011111, 11101111, 11110111, 11111011.

**Algorithm 2** Random configuration.1:**procedure** RandomConfig(c,h,d)
2: *c*: Current configuration
3: *h*: Goal Hamming distance
4: *d*: Direction: turn nodes on or off
5: $h_{max}\leftarrow h$⊳ maximum achievable Hamming distance6: $m\leftarrow \lfloor \frac{h}{2}\rfloor +1$⊳ majority7: **if**
*d* is on **then**
8:  B← number of bits set to zero in *c*
9: **else**
10:  B← number of bits set to one in *c*
11: **end if**
12: **if**
B=0
**then**
13:  c← completely random configuration
14:  **return**
*c*
15: **end if**
16: **if**
B<m
**then**
17:  $h_{max}\leftarrow 2\cdot B-1$⊳ find max. achievable18:  m←B⊳ update majority19: **end if**
20: **if**
*d* is on **then**
21:  randomly flip *m* unique bits from zero to one
22: **else**
23:  randomly flip *m* unique bits from one to zero
24: **end if**
25: $r\leftarrow h_{max}-m$⊳ remainder of possible bits to flip26: $F_{01}\leftarrow RandomNumber\left(0,r\right)$
27: a←| Randomly flip $F_{01}$ previously unflipped bits from zero to one |
28: $F_{10}\leftarrow r-a$
29: Randomly flip $F_{10}$ previously unflipped bits from one to zero
30: **return**
*c*
31:**end procedure**


### 3.3. Quick Start

The intuition with the quick start Algorithm 3 is that instead of using a completely random configuration as an initial starting point for the SNCL algorithm, a more sophisticated approach would be to quickly find a good configuration. That way, SNCL will take fewer iterations and find the best solution faster.

Specifically, Lines 3–4 set the current configuration cquick to all nodes off and assign the resulting award of that configuration to $reward_{lastIncrease}$. The for loop in Lines 5–14 first chooses a  previously unchosen node at random, sets that node to on and keeps that node on only if it results in an increased reward.

This approach does try several initial configurations (compared to just one in the completely random approach). Despite that fact, in the long term, it leads to better performance using the same number of total configuration evaluations.

**Algorithm 3** Quick start.1:**procedure** QuickStartConfiguration(*N*)2:    *N*: Number of nodes   3:    $c_{quick}\leftarrow$*N* bits all off4:    $reward_{lastIncrease}\leftarrow$ value of reward based on configuration $c_{q}uick$5:    **for** i←1 to *N* **do**6:        j← random previously unselected bit in $c_{quick}$7:        turn on *j*-th bit in $c_{quick}$8:        reward← value of reward based on configuration $c_{quick}$9:        **if** reward > $reward_{lastIncrease}$ **then**10:           $reward_{lastIncrease}\leftarrow reward$11:        **else**12:           turn off *j*-th bit in $c_{quick}$13:        **end if**14:    **end for**15:    **return** $c_{quick}$16:**end procedure**


## 4. Reward Equations

In order to evaluate the performance of the SNCL method, we have designed seven sensor network scenarios and their associated reward Equations. These reward Equations were chosen to simulate a realistic and diverse set of scenarios. In Section 5, the SNCL method is compared to several baseline methods using these scenarios.

### 4.1. Reward Equation (1): All Nodes On

Reward Equation (1) represents the scenario in which maximum application performance is achieved when all sensor nodes are on. This scenario is typical in many environments, where sensor node placement is sparse.

(1) $$R_{1}\left(c\right)=\frac{1}{HammingDistance(c_{1}^{*},c)+1}$$

where $c^{*}$ is the optimal configuration and *c* is the current configuration. For $c_{1}^{*}$, we use all nodes on.

### 4.2. Reward Equation (2): Diagonal Nodes On

**Figure 1:**
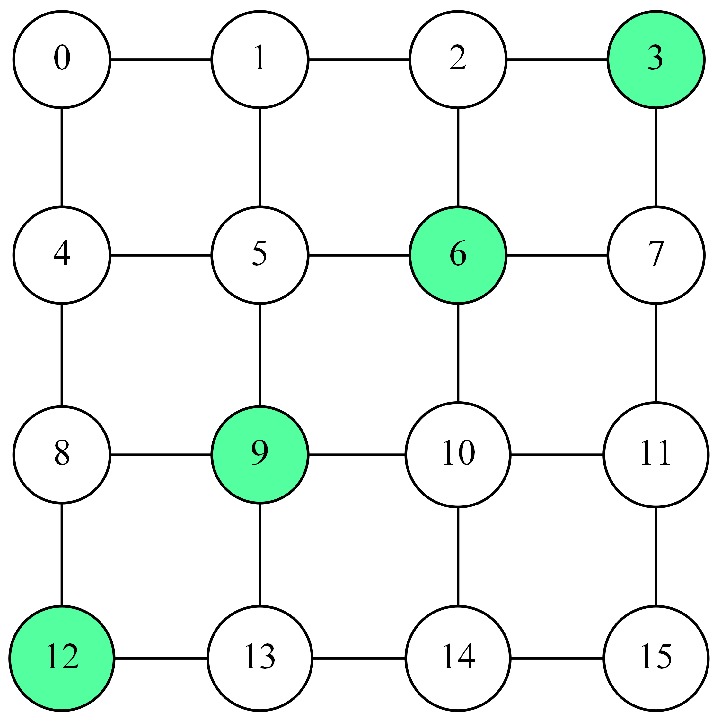
Diagonal nodes in the grid layout.

In many environments, the sensor nodes are arranged in a grid topology and designed to track the movement of an object along a certain trajectory through the environment. For this scenario, we have chosen a diagonal trajectory through a square grid topology (e.g., see Figure 1), where performance is maximized if the diagonal nodes are on and the non-diagonal nodes are off. Reward Equation (2) is similar to reward Equation (1):

(2) $$R_{2}\left(c\right)=\frac{1}{HammingDistance(c_{2}^{*},c)+1}$$

except that $c_{2}^{*}$ is defined as all diagonal nodes on and all others off. Note that the Hamming distance does not capture any notion of spatial closeness to the optimal configuration, e.g., on-nodes close to the diagonal contribute no more to performance that on-nodes far from the diagonal.

### 4.3. Reward Equation (3): Fraction of Nodes On

For many applications (though not all), the more sensor nodes that are on, the better the application’s performance. Therefore, we define reward Equation (3) as:

(3) $$R_{3}\left(c\right)=\frac{OnBits(c)}{N}$$

where OnBits(c) is the number of nodes turned on (i.e., one bits) in configuration *c* and *N* is defined as the total number of nodes in the sensor network.

### 4.4. Reward Equation (4): Non-Diagonal Nodes Off

This scenario is the same as Scenario 2 in that the only nodes we want on are those along the diagonal of a grid topology. However, in this reward Equation, we penalize nodes for being in the wrong state. Therefore, the reward Equation (4) is:

(4) $$R_{4}\left(c\right)=\left|diagNodesOn\right|-\left|diagNodesOff\right|+0.5(|nonDiagNodesOff|-\left|nonDiagNodesOn|\right)$$

The optimal configuration is when all diagonal nodes are on and all non-diagonal nodes are off. The reward Equation is calculated by adding one for each diagonal node that is on (diagNodesOn), subtracting one for each diagonal node that is off (diagNodesOff), adding 0.5 for each non-diagonal node that is off (nonDiagNodesOff) and subtracting 0.5 for each non-diagonal node that is on (nonDiagNodesOn).

### 4.5. Reward Equation (5): Distance from the Diagonal

Continuing with the tracking scenario, this scenario rewards nodes based on their distance from the track to be monitored, which again in our case will be a diagonal track through a grid topology. Specifically, the reward Equation (5) is the same as reward Equation (4) for nodes on the diagonal, but varies the non-diagonal reward/penalty from 0.1–0.5 based on the distance of the node from the diagonal.

(5) $$\begin{array}{cc} R_{5}\left(c\right)= & diagNodesOn-diagNodesOff\\ & +0.5(\sum_{nonDiagNodesOff}normDist\left(node,diag\right)-\sum_{nonDiagNodesOn}normDist\left(node,diag\right)) \end{array}$$

where normDist(node,diag) is the normalized distance from the node to the diagonal. In this case, the nodes farther from the desired track are more obviously not needed, so their state’s impact on performance is proportional to their distance from the desired track.

### 4.6. Reward Equation (6): Exactly One per Area

Many environments are arranged into well-defined areas, e.g., rooms in a home. While there may be more than one sensor in a room, there is no need for more than one of the sensors to be on. We model this scenario by dividing the *N* nodes in a grid-based sensor network into $\sqrt{N}$ rows, each with $\sqrt{N}$ nodes, where the desired configuration is exactly one node on in each row. The reward Equation is defined as:

(6) $$R_{6}\left(c\right)=\frac{1}{\left|rows\right|}\sum_{rows}\delta \;\left(OnBits\left(row\right)=1\right)$$

where δ(expr) returns one if expr is true; otherwise returns zero. The optimal configuration is determined row by row. The fractional row reward is calculated as one divided by the total number of rows in the sensor grid. Each row gets its fractional reward added to the total reward only if exactly one node is on in the row. In more complicated environments with variably-sized, irregularly-arranged areas, a similar reward Equation can be designed, but this simple arrangement captures many of the properties of such environments.

### 4.7. Reward Equation (7): Combined

**Figure 2:**
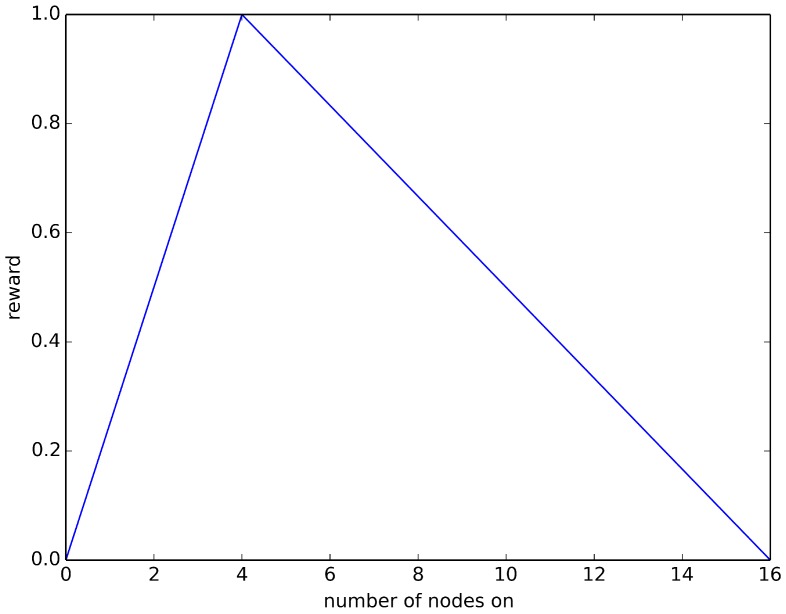
Triangle Equation for a 16-node network with opt=4.

Some scenarios require that a certain number of nodes be on, in addition to the topological arrangement of the on-nodes. To simulate this scenario, reward Equation (7) combines reward Equation (2) (nodes on the diagonal) with a “triangle” Equation that peaks at the optimal number of nodes on and linearly decreases as the number of nodes on differs more and more from optimal. Specifically, this reward Equation consists of 80% of the reward from Equation (2), plus 20% of the triangle Equation.

(7) $$R_{7}\left(c\right)=\left(0.8\right)R_{2}\left(c\right)+\left(0.2\right)Triangle\left(opt\right)$$

where opt is the number of nodes on in the sensor network configuration leading to optimal application performance. The triangle Equation is computed as follows: sum the number of on-nodes in the optimal configuration in reward Equation (2) and call that number opt; then, create a triangular Equation (e.g., Figure 2) with the *y*-axis being the reward and the *x*-axis being the number of nodes that are on in the current configuration.

The triangle Equation is constructed as follows: from x=0 to x=opt, the reward is a straight line that goes from 0–1; from x=opt to x=N, the reward is a straight line that goes from 1–0. This reward Equation is just one of many such combinations that could be tried, but is representative of such Equations.

### 4.8. Summary

The first two reward Equations use the Hamming distance, and since our inner algorithm in SNCL uses the Hamming distance, we expect that our algorithm will perform well on these Equations. They are included as a baseline to verify our algorithm is working as we expect.

The rest of the reward Equations are included to model some real-world situation. The third reward Equation models the situation where more data is better, and the maximum reward is obtained when all nodes are on.

The fourth reward Equation could be a situation where a road goes through an area that needs to be monitored and any nodes on that are not on the road subtract equally from the reward. The fifth reward Equation is the same as the fourth except the on-nodes that are not near the road subtract from the reward proportional to their distance from the road (the farthest ones away are the ones we want off the most).

The sixth reward Equation could be envisioned as a home or office building that is divided up into rooms, where the rows in our grid layout map to rooms. Then, each room gets its fractional reward only if exactly one node is on.

The seventh reward Equation uses a triangular Equation and simulates the situation where starting out adding nodes helps increase the reward, as more nodes results in obtaining more data. However, after some point, the cost of adding additional nodes is detrimental (e.g., when the energy consumption of the nodes is taken into consideration).

For reward Equations (1) and (3), no assumptions are made as to the topology of the network. A grid topology is assumed in reward Equations (2) and (4)–(7); however, the grid topology is only needed in order to define a topologically-dependent reward Equation. Other topologies can be considered as long as topologically-relevant reward Equations can be defined. Our method can be applied to any topology, but is designed to exploit topological patterns in typical real-world deployments.

## 5. Experiments

We performed the experiments by using the ns-3 sensor network simulator (https://www.nsnam.org) to evaluate our algorithms [6] and compare them with the probabilistic selection [8] sensor selection algorithm, genetic algorithm and pure randomness (use of a different random configuration on every iteration) under the scenarios listed in Section 4. By iteration, we mean a complete evaluation cycle of the algorithm: set the configuration, then wait for the reward obtained using that configuration.

Simulations were run on 5×5, 7×7 and 10×10 grid-shaped topologies with total node counts equaling 25, 49 and 100, respectively. The parameter *T* was experimentally determined by running until SNCL without quick start found a configuration yielding the maximum application performance. All experiments were run 10 times. The average values of the results are presented. Our experiments were run using an increasing number of nodes to test the scalability of the SNCL algorithm.

Our idea for reconfiguring the network is as follows: the sink node listens for data packets and, once per minute, broadcasts a reconfiguration packet that contains the on/off status for each node. All nodes listen for reconfiguration packets for 3 s out of every minute. The 60-s and 3-s values for these settings are estimates based on our experience. They can be tuned for each specific deployment.

A sensor network with a large number of nodes would probably require a different way of reconfiguring. There are other possible schemes that might work on a large network besides flooding, like dividing the network up into regions. That way, each region would be retransmitting a much smaller regional configuration string (thereby requiring a smaller window for each node to capture the reconfiguration message); or a more energy-efficient protocol could be used, like a gossip protocol.

### 5.1. Baseline Algorithm

We include a comparison against a standard genetic algorithm as a baseline method for evaluating our SNCL algorithm. Genetic algorithms (GAs) are heuristic search algorithms based on the idea of natural selection. They were popularized by [18] at the University of Michigan.

Our implementation of the genetic algorithm uses the following standard parameters:

- Population size is set to the number of nodes in the sensor network
- Parents chosen by being randomly weighted based on fitness
- Single crossover point method
- Probability of crossover = 0.60
- Probability of mutation = 0.01

We ran experiments using different parameter settings for the genetic algorithm. Specifically, we tried different population sizes (*N*, N×10), different crossover probabilities (0.6, 0.7, 0.8, 0.9) and different mutation rates (0.01, 0.1, 0.25). The results from these additional runs did not produce significantly better performance than the standard parameter settings above.

First, we create the initial population with random configurations. These individuals get evaluated by running them and obtaining the reward associated with using them as a configuration, which is called their fitness. To create the next generation, the individuals are ranked by fitness. Looping over starting from the highest fitness, we breed them with 60 percent probability, otherwise they get passed on without crossover. A single crossover point is randomly picked, and two individuals are created by swapping at the crossover point. After the mating is done, with probability 0.01, a single bit of the individual configuration is changed. Then, the individuals are evaluated for fitness, and then, next generation is created.

### 5.2. Simulation Results

**Figure 3:**
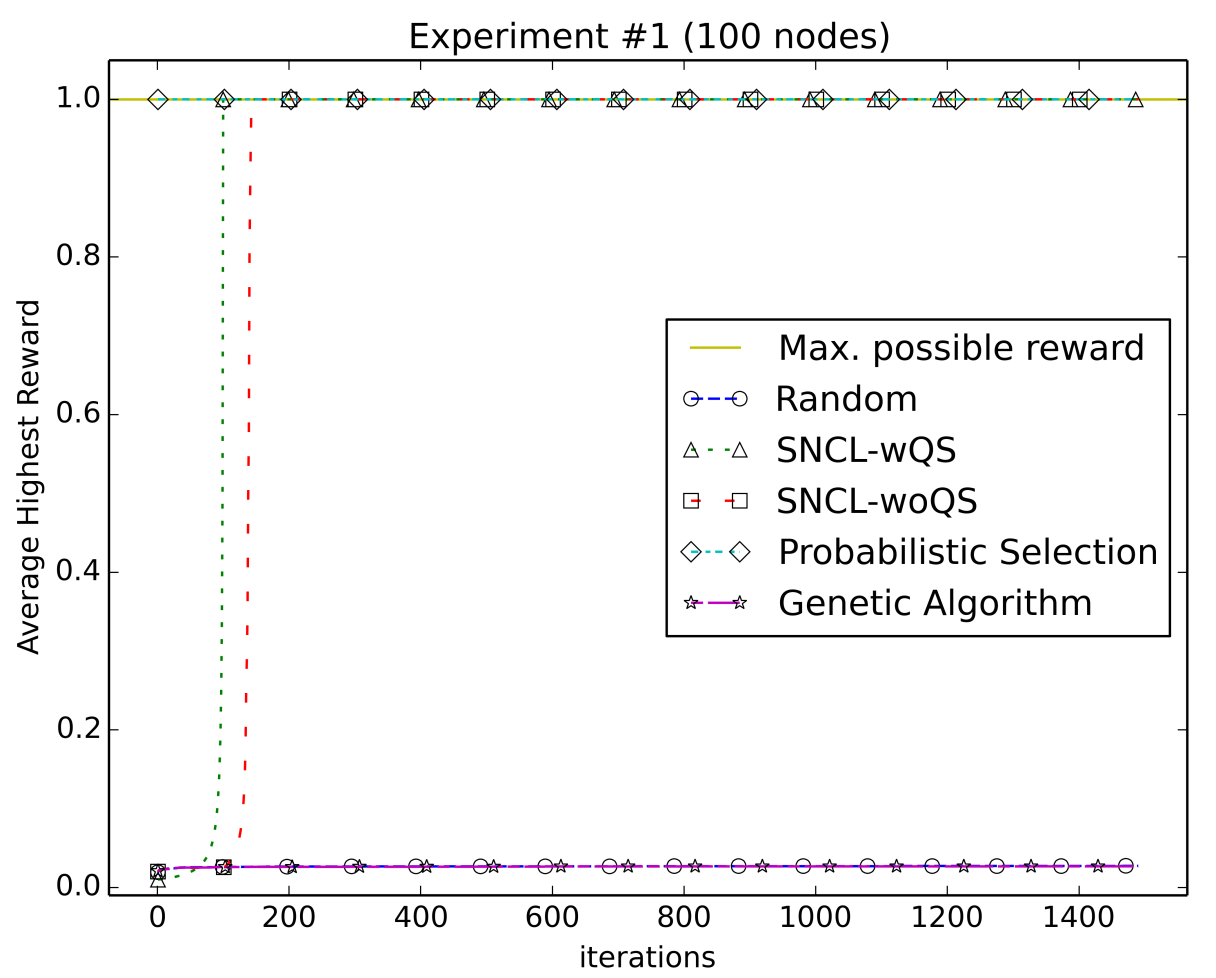
Using reward Equation (1). SNCL-woQS, sensor network configuration learning without quick start.

**Figure 4:**
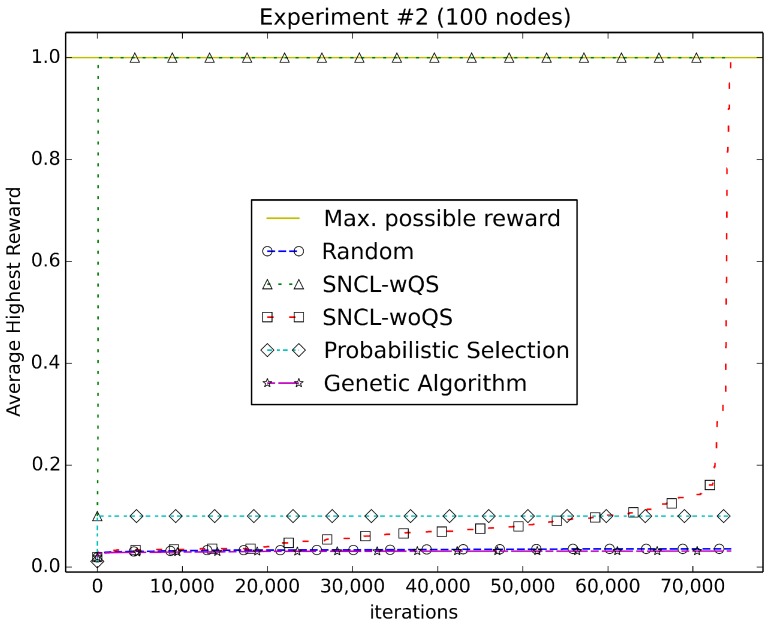
Using reward Equation (2).

**Figure 5:**
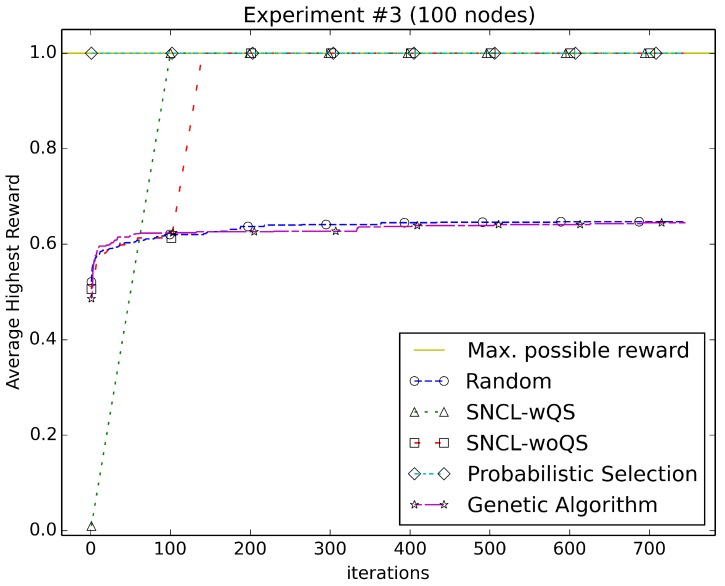
Using reward Equation (3).

**Figure 6:**
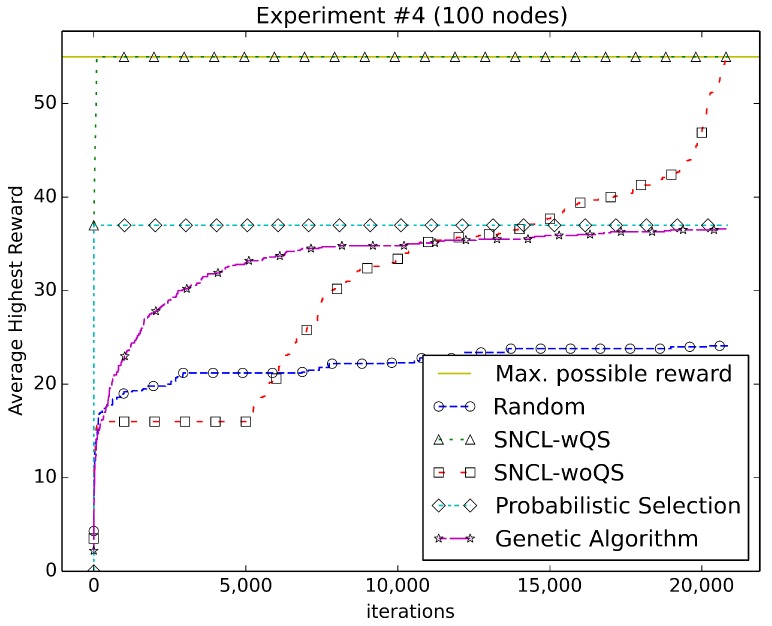
Using reward Equation (4).

**Figure 7:**
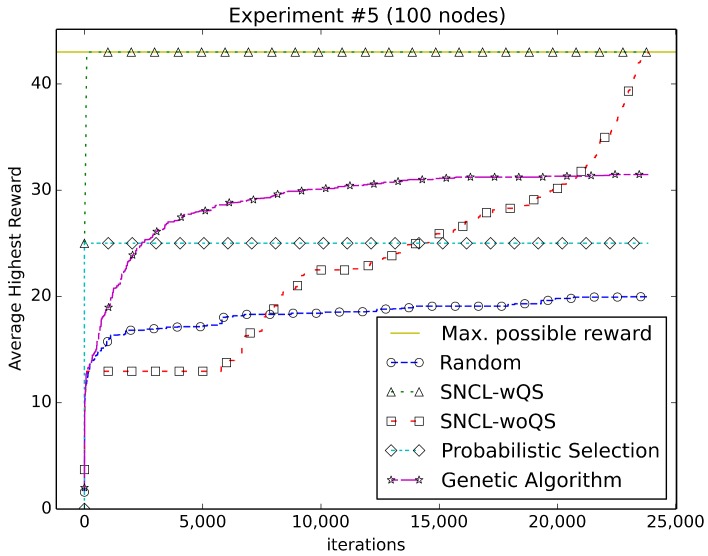
Using reward Equation (5).

**Figure 8:**
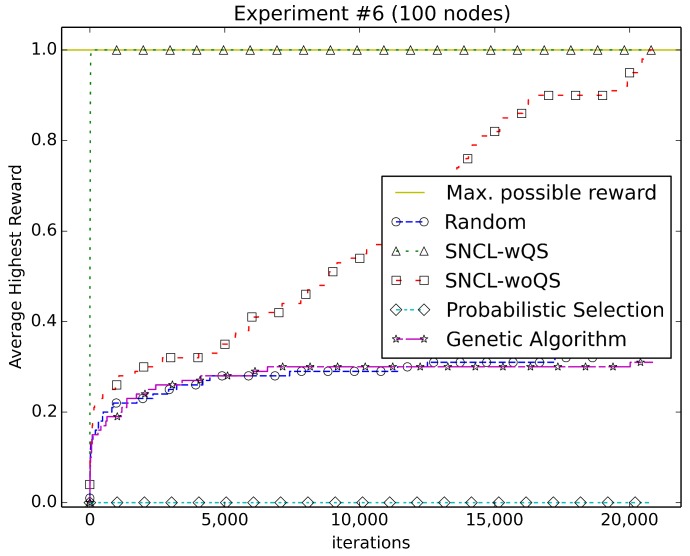
Using reward Equation (6).

**Figure 9:**
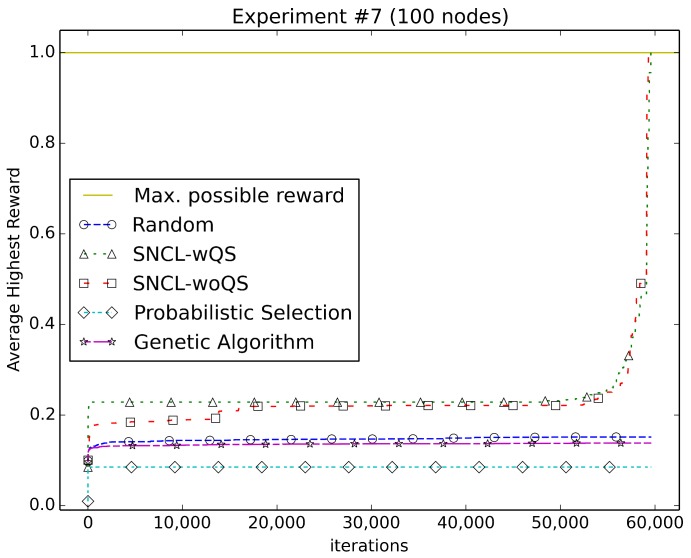
Using reward Equation (7).

Figure 3, Figure 4, Figure 5, Figure 6, Figure 7, Figure 8 and Figure 9 compare the performance of the approaches using the main algorithm augmented with (SNCL-wQS) and without (SNCL-woQS) the quick start option versus probabilistic selection, genetic algorithm and pure randomness.

The reason for running experiments without quick start is to demonstrate how the algorithm performs assuming that the quick start algorithm does not return a good initial configuration. In that case, a random configuration is selected for the initial configuration.

In all the figures, the *x*-axis shows the number of iterations and the *y*-axis the highest reward seen so far.

Experimenting with reward Equation (1) (Figure 3), PSfinds the highest reward possible of 1.0 on the first iteration due to the PS algorithm trying the all-nodes-on configuration first. The quick start part of the SNCL algorithm quickly finds the highest reward at 100 iterations; without quick start is not far behind by finding the highest reward at 143 iterations. the genetic algorithm roughly does the same as randomness during the entire time, with both never obtaining better than 0.03 for the highest reward seen.

With reward Equation (2) (Figure 4), SNCL finds the highest reward of 1.0 very fast at 100 iterations, while SNCL without quick start finds the highest reward at 74,435 iterations. PS gets 0.1 for the highest. Randomness does slightly better than the genetic algorithm, but both are close to 0.03 at the finish.

With reward Equation (3) (Figure 5), PS instantly finds the highest reward of 1.0 due to trying the all-nodes-on configuration as a first step. SNCL finds the highest reward after executing the quick start algorithm, and without quick start quickly ramps up to the highest reward at 142 iterations. Randomness and the genetic algorithm do roughly the same, and both finish at around 0.64 for the highest.

With reward Equation (4) (Figure 6), SNCL finds the highest reward of 55 after completing quick start; without quick start, it finds the highest around 20,000 iterations. PS finds the highest reward of 37. The genetic algorithm almost matches with a 36.6 highest reward. Randomness does the worst finishing at 24.

With reward Equation (5) (Figure 7), SNCL finds the highest reward of 43 right after completing quick start. SNCL without quick start does slightly worse than the others at the start, but then improves, ramping up to find the highest at 23,834 iterations. The genetic algorithm does better than PS and randomness, but levels off around 31. PS finds 25 for the highest, and randomness never gets over 20.

With reward Equation (6) (Figure 8), SNCL finds the highest reward of 1.0 during quick start. SNCL without quick start has mixed results early on, but it then finds the highest at 20,752 iterations. The genetic algorithm and randomness do roughly the same and end up around 0.3 for the highest. PS never does better than zero for the highest reward seen due to the assumption that the configuration with all nodes on will have the best reward, which is obviously not true in this case.

With reward Equation (7) (Figure 9), SNCL both with and without quick start performs considerably better than the other algorithms. Both SNCL algorithms find the highest reward possible of 1.0 around 59,000 iterations. Randomness, the genetic algorithm and PS end up at 0.15, 0.08 and 0.13, respectively.

### 5.3. Scalability

The scalability is determined by the number of iterations it takes to find the maximum reward vs. the number of nodes in the network. We ran simulations on networks having 25, 49 and 100 nodes.

**Figure 10:**
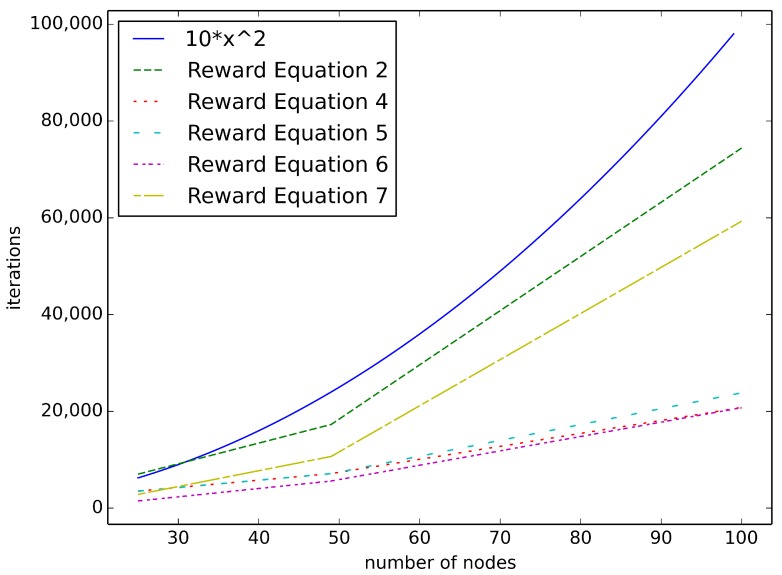
Scalability of SNCL without quick start.

For SNCL without quick start, both Experiments 1 and 3 have linear scalability. The rest of the experiments are shown in Figure 10.

For SNCL with quick start, all of the experiments except Experiment 7 show roughly linear scalability. Experiment 7 with quick start has results almost identical to Experiment 7 without quick start (see Figure 10).

## 6. Discussion

In all of the 100-node experiments, SNCL performs better than pure randomness in that it finds the highest reward possible before randomness. In fact, randomness is never able to find the highest reward in any of the scenarios.

When SNCL does not use the quick start option (or when quick start does not find a good initial configuration as with reward Equation (7)), it is able to eventually find the highest reward possible, something PS does not do. PS sometimes finds the highest slightly faster than SNCL; however, if PS does not immediately find the highest, then it will stay at a suboptimal reward level.

In most cases, the genetic algorithm does roughly the same as randomness or slightly better. GA does seem to be better than PS except for the times in which, by coincidence, PS gets the highest reward on the first try by trying all nodes on at the first step. Both GA and randomness are never able to find the highest reward in any of these scenarios. There are several reasons why GA performs poorly on these tasks. First, compared to random crossover and mutation, using Hamming distance in the direction of improvement as the guidance for generating new configurations is better for typical sensor network configurations. Second, the linearly-decreasing Hamming distance limit helps SNCL to make several small changes at the end of the exploration time window; whereas GA has no concept of “closeness” of neighbor configurations and therefore cannot derive any benefit from that in its algorithm. Third, each new generation of *N* individuals in GA implies the need to evaluate *N* different configurations of the sensor network, each of which takes some time, so GA in general will take much longer to reach a specific level of performance. Fourth, the QuickStart method gives SNCL a good starting point; GA could also be seeded with this starting point, but would then not be a pure GA approach and would likely still perform worse due to the above issues.

Overall, our experiments show that SNCL with or without quick start performs better than the genetic algorithm, randomness and the PS algorithm using a variety of reward Equations. PS can sometimes find the highest reward possible slightly faster than SNCL because as a first step, it tries all nodes on; however, with many reward Equations, it finds a much lower highest reward. If the SNCL quick start option does not find a good configuration to start with, we show that the main SNCL algorithm will eventually find the highest reward possible.

To see how our algorithm scales, we ran it on networks having 25, 49 and 100 nodes. The SNCL algorithm, both with and without QuickStart, in our experiments shows polynomial (low-order quadratic) scalability in the worst case.

## 7. Conclusions

This paper describes a novel method for dynamically finding the best WSN configuration, within the allowed time, that will maximize the performance of the target application. Because different scenarios may have unique reward Equations, our algorithm was run on a wide range of scenarios. Our results show that the SNCL heuristic algorithm outperforms the other techniques investigated, such as the standard genetic algorithm, probabilistic selection and pure randomness. Our results show that SNCL scales well; in the worst case, it shows low-order quadratic polynomial scalability. SNCL with quick start is the best approach because it can easily find a good solution in most applications, and the reward Equations are near monotonic. The SNCL approach could be applied to many applications such as activity recognition while minimizing power consumption in a home or indoor building environment.

There is potential for the work to be extended in many different ways. Variable data rates and mobile nodes could be taken into consideration. Experimental evaluation of our algorithm could be conducted on real data collections. Additionally, other heuristic methods could be incorporated such as Tabu search or particle swarm optimization.

## Figures and Tables

**Table 1:** Algorithm definitions.

Symbol	Definition
*N*	Number of nodes in the WSN
*T*	Number of exploration iterations
*H*	Initial Hamming distance, where 1≤H≤N
*h*	Dynamic Hamming distance used to obtain neighbor configurations, which linearly decreases from *H*–1
*c*	Configuration (bit string)
*i*	Iteration number
OnBits(c)	Returns the number of bits set to 1 in configuration c
